# The association between peer relationship and learning engagement among adolescents: The chain mediating roles of self-efficacy and academic resilience

**DOI:** 10.3389/fpsyg.2022.938756

**Published:** 2022-08-03

**Authors:** Yanhong Shao, Shumin Kang

**Affiliations:** ^1^Faculty of Education, Qufu Normal University, Qufu, China; ^2^Jiangsu Xiangshui Senior High School, Yancheng, China; ^3^College of Foreign Languages, Qufu Normal University, Qufu, China

**Keywords:** peer relationship, learning engagement, self-efficacy, academic resilience, adolescents

## Abstract

Previous studies have shown that peer relationship affects learning engagement. And learning engagement plays a vital role in promoting knowledge acquisition and production, enhancing adolescents’ academic success. However, few studies have focused on the mechanism between peer relationship and learning engagement. As such, based on Social Cognitive Theory, this study attempts to explore how peer relationship of adolescents is linked to learning engagement through the chain mediating roles of self-efficacy and academic resilience. The participants were 250 students who were selected *via* random sampling in a public middle school, in Eastern China, in June 2021. All the participants filled in the structured self-report questionnaires on peer relationship, self-efficacy, academic resilience, and learning engagement. The data were analyzed with structural equation modeling (SEM) in SPSS 24.0 and AMOS 24.0. Results indicated that peer relationship was directly and positively associated with learning engagement. Results also indicated that peer relationship was indirectly and positively associated with learning engagement *via* self-efficacy and academic resilience, respectively, and sequentially. More importantly, it was found that the direct effect was much lower than the indirect effects of which self-efficacy was the greatest. It is suggested that appropriate interventions and support should be provided to facilitate adolescents’ peer relationship, self-efficacy, and academic resilience, thus promoting their learning engagement and academic success.

## Introduction

Peer relationship refers to a kind of interpersonal relationship developed in the process of interaction in small clusters of individuals that are closely connected with each other based on shared interests and friendships (Rohrbeck and Garvin, 2014). Peer relationship is categorized into dimensions such as warmth, support, attachment, friendship quality, and communication quality (Boele et al., 2019; Terlektsi et al., 2020). As a critical social relationship, peer relationship is crucial to the physical and mental development of adolescents. It not only reduces adolescents’ social anxiety, shapes their moral cognition and behaviors, but also enhances their engagement, which contributes to their academic successes (Fredricks, 2011; Tillfors et al., 2012; Zulfiqar, 2020; Chiu et al., 2021). Student engagement, as a key element in learning, can be defined from three perspectives, namely, behavioral, emotional, and cognitive engagement (Fredricks et al., 2004; Reeve and Tseng, 2011; Yazzie-Mintz and McCormick, 2012). Behavioral engagement refers to students’ participation and involvement in academic activities that reflect on-task attention, effort, and persistence (Fredricks et al., 2004). Emotional engagement refers to student’s positive feeling, attitude, and perception toward learning activities (Park and Yun, 2017; Tvedt et al., 2019). Cognitive engagement refers to students’ active involvement in learning with positive psychological status (Nguyen et al., 2016; Yang Y. et al., 2021). Among them, behavioral engagement reflects the substantive connotation of student engagement (Newmann, 1992) and it is relatively easier to measure due to their observable characteristics (Nguyen et al., 2016). Based on the above literature, learning engagement can be defined as students’ positive psychological state of mind concerning learning behaviors, with three dimensions—vigor, dedication, and absorption (Schaufeli et al., 2002a; Christenson et al., 2012). Vigor is defined as how individuals are ready to work hard and persevere in their studies, even in facing difficulties. Dedication refers to individuals’ strong senses of responsibility and achievement toward learning, while absorption refers to individuals’ concentration on learning for long periods of time and obtaining positive psychological experiences during the process of learning (Li et al., 2019).

Research has shown that peer relationship is correlated with learning engagement, in which self-efficacy is a potential predictor (Sökmen, 2019). Self-efficacy is understood as “an individual belief in one’s capabilities to organize and execute the courses of action required in producing given attainments” (Bandura, 1997a, p3). It is also defined as “the perception of one’s ability to successfully perform a particular behavior” (Block et al., 2010, p44). Research has noted that academic resilience is also a potential predictor of learning engagement (Romano et al., 2021). Academic resilience is considered as the personal ability to overcome acute or chronic adversity in learning (Martin, 2013) or effectively deal with setbacks, challenges, adversity, and pressure in the academic setting (Martin and Marsh, 2006) with three-dimensional elements, namely, perseverance, adaptability, and emotional response (Cassidy, 2016). However, few studies have tested how peer relationship of adolescents is linked to learning engagement through the mediating roles of self-efficacy and academic resilience based on relative theory.

Social Cognitive Theory (SCT) (Bandura, 1997b) is based on a psycho-social model, which explains socio-cognitive constructs of behaviors (Komendantova et al., 2018). It has been viewed as an important theoretical framework to explain human behaviors (Yazdanpanah et al., 2015; Hou et al., 2021). SCT proposes that environment and personal factors influence human behaviors (Bandura, 1986). That is to say, human behaviors are motivated and regulated by a combination of environmental, personal, and behavioral factors (Bandura, 2012). Environmental factors are social support and barriers to individuals’ behaviors. Personal factors include knowledge, self-efficacy, and outcome expectations associated with behavioral adoption (Komendantova et al., 2018). Of the personal factors, self-efficacy is a major element and plays a central role in changing behaviors (Bandura, 1997b). Behavioral factors consist of endeavor or planning to execute a behavior (Shahangian et al., 2021). Several researchers have applied SCT to explore classroom cognitive engagement or online learning engagement among college students (Sahil and Hashim, 2011; El-Sayad et al., 2021; Kuo et al., 2021). However, little has been done to explore the interrelated associations of the influencing factors in adolescents’ learning engagement with SCT. Therefore, the study attempts to apply SCT, (1) to explore the mechanism in which peer relationship predicts learning engagement among adolescents *via* self-efficacy and academic resilience, and (2) to provide evidence for how peer relationship influences adolescents’ learning engagement.

The study includes the following contributions. First, the study examines the association between peer relationship and learning engagement based on Social Cognitive Theory in the Chinese context, which provides evidence for the research on similar themes in other countries. Second, the study explores the mechanism between peer relationship and learning engagement by emphasizing the chain mediating roles of self-efficacy and academic resilience. The new perspective may explain that adolescents’ learning engagement is mainly affected by self-efficacy and academic resilience (personal factors) that stem from sound peer relationship (environmental factor).

### Peer relationship and learning engagement

Relevant studies have showed that peer relationship can exert a direct influence on learning engagement (Juvonen et al., 2012; Gremmen et al., 2018). Fredricks et al. (2019) have suggested that support from peers aligns with greater learning engagement. Similarly, Kiefer et al. (2015) have pointed out that support from peers can exert a profound influence on students’ learning engagement. When students can get support from their peers, they are more likely to feel confident in learning; on the contrary, when students have less support from their peers, they are more likely to feel afraid to accomplish tasks, which lessens their learning engagement (Juvonen et al., 2012; Geven et al., 2013; Shin and Chang, 2022). In addition, Furrer et al. (2014) have reported that the quality of students’ relationships with peers is a fundamental substrate for the development of learning engagement. It is reported that high-quality friendship is protective against being conflicted, rejected, and bullied, which promotes engagement in learning (Terlektsi et al., 2020). Hence, it could be argued that adolescents with sound peer relationship are likely to engage in learning. Based on this view, the following hypothesis is proposed.

H1: Peer relationship is positively associated with learning engagement.

### Self-efficacy as a mediator

In social cognitive theory, Bandura (1997b) has emphasized the construction of self-efficacy and its impact on learning. Students with stronger self-efficacy tend to set higher goals and undertake more challenging tasks. And they are more likely to put forth the effort and be persistent in learning. Even when it comes to academic challenges or difficulties, they still stick to it instead of giving it up (Masud et al., 2016).

Several studies have acknowledged that self-efficacy is often influenced by peer interaction (Ruegg, 2014; Sökmen, 2019; Shyr et al., 2021). Support from peer interaction is important in establishing a positive attitude and increasing self-confidence and the ability to make judgments in learning (Chu and Chu, 2010), while imitation from peer interaction contributes to the development of adolescents’ cognition, emotion, and behaviors. It is reported that adolescents accept the influence of role models in peer imitation to promote the development of their self-efficacy (Lee et al., 2021). In addition, peer collaboration exerts an influence on self-efficacy (Lee and Evans, 2019). It is believed that peer relationship is positively associated with adolescents’ self-efficacy.

Self-efficacy is also believed to be one of the key factors influencing students’ learning engagement (Wu et al., 2020; Shao and Kang, 2022). Students with higher self-efficacy have higher engagement in learning. Some researchers have suggested that self-efficacy can help develop positive beliefs about personal skills and abilities, thus enabling students to be more involved in their learning (Zhen et al., 2017; Ahmed et al., 2018). Other researchers have argued that self-efficacy affects students’ classroom participation, thereby affecting students’ learning engagement (Sökmen, 2019). Similarly, Liem et al. (2008) have also pointed out that peer relationship plays an important role in adolescents’ self-efficacy, which affects their learning engagement. The above views indicate that peer relationship may affect adolescents’ learning engagement *via* the indirect role of self-efficacy. Based on these, the following hypotheses are proposed:

H2: Peer relationship is positively associated with self-efficacy.

H3: Self-efficacy is positively associated with learning engagement.

H4: Self-efficacy plays a mediating role in the association between peer relationship and learning engagement.

### Academic resilience as a mediator

Academic resilience is influenced by peer relationship (Baltaci and Karataş, 2015). Permatasari et al. (2021) have proposed that peer support could contribute to academic resilience in the learning process. Chen et al. (2017) have emphasized that peer support was a consistent predictor of academic resilience. Hoshek et al. (2016) have argued that more contact with peers can ease students’ negative perceptions in dealing with academic challenges. Frisby et al. (2020) have also argued that the relational resources that students have at school, especially with peers, may inspire students’ academic resilience. Hence, these shreds of evidence support the belief that peer relationship may enhance adolescents’ academic resilience.

Academic resilience influences adolescents’ learning engagement (Cheung et al., 2014). Students with academic resilience tend to express higher levels of achievement despite risks and difficulties (Simões et al., 2021). Romano et al. (2021) have argued that students with a higher level of academic resilience show a higher level of learning engagement. Gillham et al. (2013) have demonstrated that students who feel more connected with peers have higher academic resilience, which plays a crucial role in learning engagement. Therefore, this study speculates that there is a positive relationship between adolescents’ academic resilience and their learning engagement, and academic resilience may play an intermediary role between peer relationship and learning engagement.

Academic resilience is believed to influence by self-efficacy (Cassidy, 2016). In another word, self-efficacy is a significant predictor of academic resilience (Martin and Marsh, 2008; Murray, 2018; Rachmawati et al., 2020; Hydar Choupani and Dehsorkhi, 2021; Kuo et al., 2021), which provides a fundamental basis for the serial variables of self-efficacy and academic resilience. According to SCT, the environment filled with peers is conducive to enhancing their self-efficacy (Zysberg and Schwabsky, 2020). With enhanced self-efficacy, students are more able to encounter difficulties, engage themselves in challenging learning tasks, and develop their academic resilience (Skinner and Pitzer, 2012; Cassidy, 2015). And the personal factors—self-efficacy and academic resilience affect their behavior—learning engagement (Wang and Holcombe, 2010; Honicke and Broadbent, 2016; Vîrgã et al., 2020). Therefore, it is believed that peer relationship may influence learning engagement *via* the serial variables of self-efficacy and academic resilience.

Based on the above analysis, this study intends to examine whether peer relationship may positively contribute to learning engagement *via* the mediating roles of sequential self-efficacy and academic resilience. In view of this, the following hypotheses are proposed:

H5: Peer relationship is positively associated with academic resilience.

H6: Self-efficacy is positively associated with academic resilience.

H7: Academic resilience is positively associated with learning engagement.

H8: Academic resilience plays a mediating role in the association between peer relationship and learning engagement.

H9: Self-efficacy and academic resilience play a chain mediating role in the association between peer relationship and learning engagement.

**FIGURE 1:**
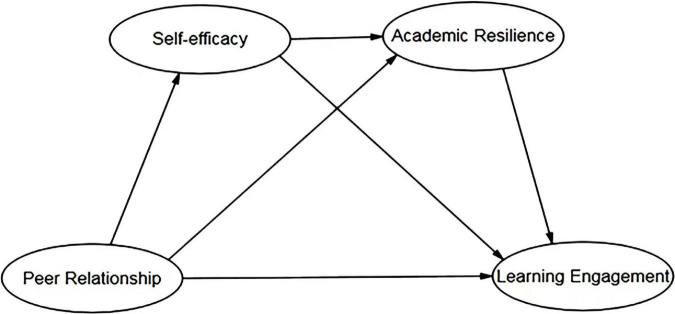
The Proposed theoretical model.

Guided by Social Cognitive Theory and the above hypotheses, we have constructed a theoretical model to test the association between peer relationship and learning engagement, as well as the mediating roles of self-efficacy and academic resilience (see Figure 1).

## Materials and methods

### Sampling and procedure

The sample size was estimated according to the requirement of Structural Equation Modeling (SEM) (Zhang et al., 2020) that the appropriate sample size was targeted at least ten times the total observed variables. The samples for the study were drawn from participants who were 13–14 years old from a public middle school, in Eastern China, in June 2021. One of the main reasons for choosing the school was that it is a relatively large-scale public school with more than 3,000 students. In the school, 270 students from seventh and eighth grades were randomly chosen to participate in the survey. Finally, 250 valid samples with a response rate of 92.6% were obtained and adopted for data analysis.

Before conducting the study, permission was obtained from the Research Ethics Committee of Qufu Normal University, the headmaster of the participating school, and the parents of the participants. Then, the survey was described to the students for a better understanding. Lastly, the students were told the purpose of the study and guided to complete the questionnaires anonymously.

### Questionnaire design

The questionnaire was designed with reference to previous instruments that had been widely accepted with high reliability and validity. It was composed of two main parts. The first part aimed to measure the general demographic variables to capture sample characteristics. The second part, as the main body of the questionnaire, consisted of four latent variables, namely, peer relationship, self-efficacy, academic resilience, and learning engagement, with nineteen measurement items (Table 1). All measurement items within the model were rated on a 5-point Likert scale with a response category ranging from 1 (strong disagreement) to 5 (strong agreement). The four dimensions of the questionnaire were modified from well-accepted instruments. The four items of peer relationship were from Wei (1998). The five items of self-efficacy were from Schwarzer (1994). The five items of academic resilience were from Cassidy (2016). The five items of learning engagement were from Fang et al.’s (2008) Chinese version modified in line with Utrecht Work Engagement Scale-Student (Schaufeli et al., 2002a,b). The modified items had good reliability and validity in the context of Chinese culture, which has been widely used in China. The specific measurement items are shown in Table 1.

**TABLE 1:** Latent variables and items.

Latent variable	Code	Measurement items
Peer relationship (PR)	PR1	Classmates are willing to listen to my opinions.
	PR2	When classmates are ill, I feel very sad.
	PR3	When I achieve success, my classmates are proud of me.
	PR4	When classmates are unhappy or crying, I usually go to comfort them.
Self-efficacy (SE)	SE1	If I try my best, I can always solve problems.
	SE2	It is easy for me to pursue my dream and achieve my goals.
	SE3	I can calmly face difficulties because I trust my ability to deal with problems.
	SE4	When there is trouble, I can usually think of some ways to cope with it.
	SE5	No matter what happens to me, I can handle it.
Academic resilience (AR)	AR1	When facing difficulties in learning, I can try to think of new solutions.
	AR2	When I am discouraged by my studies, I can use situations to motivate myself.
	AR3	I can’t change my long-term goals and ambitions until I make a success.
	AR4	I usually look forward to showing that I can improve my grades.
	AR5	I can do my best to stop thinking negative thoughts when I fail to achieve the desired goals.
Learning Engagement (LE)	LE1	When I get up in the morning, I want to study.
	LE2	I can keep on learning, even if it does not go smoothly.
	LE3	I feel that I have a clear learning goal and that learning is meaningful.
	LE4	When I study, I feel time passing quickly.
	LE5	I am proud of my persistent learning.

### Statistical analysis

The data were analyzed with SPSS 24.0 and Amos 24.0. First, the Harman single factor test was carried out to test the common method bias. Then, descriptive analysis was conducted to examine the sample characteristics. Finally, structural equation modeling (SEM) analysis was performed to examine the measurement model and the structural model. Specifically, confirmatory factor analysis was performed to examine the reliability and validity by providing the values of factor loadings, CR, and AVE. And the analyses of the goodness-of-fit index and path coefficient were adopted to test the acceptable level for the structural model. In addition, sensitivity analysis was conducted to calculate the effect size. Lastly, the bootstrapping method was used to evaluate the statistical significance of the mediating effects of the proposed hypotheses.

## Results

### Common method variance

All the data were obtained from the self-report of middle school students. In order to reduce the common method variance that may influence the validity and reliability of the study (Podsakoff et al., 2012), the Harman single factor test was adopted to test the common method bias by SPSS 24.0 (Podsakoff et al., 2003). The results indicated that there were 4 factors with a characteristic root greater than 1, and the variance explanation rate of the first factor was 41.696%, less than the critical criterion of 50% (Hair et al., 2010), indicating that the common method variance was not serious.

### Sample characteristics

As shown in Table 2, the distribution between males and females was almost equal. The sample was split evenly across gender, with 48% of students studying in Grade Seven and 52% in Grade Eight. Students living in the towns were the larger group in the sample. Students were split across median household monthly income with a great proportion falling from 5,000 to 10,000 Yuan (42%), 3,000–5,000 Yuan (38%), less than 3,000 Yuan (11.6%) to 10,000 Yuan and more (8.4%).

**TABLE 2:** Descriptive summary of socio-demographic profile of students.

Demographic	Sample (*n* = 250)	Frequency	Percentage
Gender	Male	117	46.8%
	Female	133	53.2%
Grade	Grade 7	120	48%
	Grade 8	130	52%
Resident	Town	198	79.2%
	Countryside	52	20.8%
Median household monthly income	Less than 3,000 Yuan	29	11.6%
	3,000–5,000 Yuan	95	38.%
	5,000–10,000 Yuan	105	42.%
	10,000 Yuan and more	21	8.4%

### Measurement model

The study aimed to test the measurement model with CFA by reporting the reliability and validity of the model. Cronbach’s α is used as the most common index to estimate the reliability. Its value ranges between 0.80 and 0.89, indicating that the model is reliable (Yockey, 2010). Factor loadings, composition reliability (CR), and the average variance extracted (AVE) are adopted to measure convergent validity (Chen and Lin, 2019). All the indexes are 0.5 or higher, indicating this model has good convergent validity. The square root value of AVE is greater than the correlation coefficient value, showing that there is discriminant validity between the constructs (Fornell and Larcker, 1981).

As indicated in Table 3, Cronbach’s α ranged from 0.818 to 0.901. The standardized factor loadings ranged from 0.671 to 0.864 and they were significant (*p* < 0.001). The values of CR and AVE ranged from 0.820 to 0.903, and from 0.533 to 0.651 respectively. It can be seen from Table 4 that the square root values of AVE in each construct were greater than any other correlation coefficient value. Overall, all the values exceeded the standardized value, thus indicating that the model had a reasonable degree of reliability and validity.

**TABLE 3:** Reliability and validity examination.

Latent variable	Item	UC	SE	Z-value	*P*-value	SC	Cronbach’s a	CR	AVE
Peer relationship (PR)	PR1	1.000				0.705			
	PR2	0.985	0.101	9.731	***	0.703			
	PR3	1.176	0.108	10.896	***	0.829	0.818	0.820	0.533
	PR4	0.939	0.100	9.377	***	0.674			
Self-efficacy (SE)	SE1	1.000				0.793			
	SE2	0.910	0.071	12.907	***	0.761			
	SE3	1.033	0.068	15.148	***	0.864	0.901	0.903	0.651
	SE4	1.011	0.074	13.682	***	0.797			
	SE5	0.985	0.070	14.099	***	0.816			
Academic resilience (AR)	AR1	1.000				0.706			
	AR2	1.024	0.099	10.383	***	0.722			
	AR3	1.223	0.105	11.680	***	0.827			
	AR4	1.064	0.101	10.533	***	0.734	0.850	0.853	0.539
	AR5	1.069	0.110	9.695	***	0.671			
Learning engagement (LE)	LE1	1.000				0.707			
	LE2	1.186	0.103	11.513	***	0.786			
	LE3	1.250	0.103	12.097	***	0.831	0.877	0.878	0.592
	LE4	1.165	0.108	10.787	***	0.734			
	LE5	1.231	0.107	11.467	***	0.783			

UC, Unstandardized Coefficients; SE, standard error; SC, standardized coefficients.

***p < 0.001.

**TABLE 4:** The discriminate validity test of latent variables.

Latent variable	Peer relationship	Self-efficacy	Academic resilience	Learning engagement
Peer relationship	**0.730**			
Self-efficacy	0.437***	**0.807**		
Academic resilience	0.441***	0.557***	**0.734**	
Learning engagement	0.514	0.646	0.663	**0.769**

The square root of the AVE of four latent constructs is given in the diagonal, and the correlation coefficient is given on the below diagonal.

The bold values represent the square root of AVE.

***p < 0.001.

### Structural model

The study adopted the goodness-of-fit index and path coefficient to assess the structural model in Amos 24.0. Researchers suggested that a structural model had a good fit to the data with indexes of x^2^/df (Chi-square/df) between 0 and 3, IFI, CFI, TLI, GFI, and AGFI greater than 0.9, SRMR and SMSEA less than 0.08 (Zhang et al., 2020). Table 5 shows that their goodness-of-fit index values were as follows: Chi-square (X^2^)/df = 1.469 (X^2^ = 214.446, df = 146), IFI = 0.973, CFI = 0.972, TII = 0.968, GFI = 0.914, AGFI = 0.888, SRMR = 0.0483, SMSEA = 0.043. The result of sensitivity analysis also shows that the effect size was 0.437, reaching the cut-off value of effect size that Cohen (1992) recommended. As such, the current 250 sample size can obtain statistically convincing test results.

**TABLE 5:** Goodness of fit index of the structural model.

Fit index	X^2^/df	IFI	CFI	TLI	GFI	AGFI	SRMR	SMSEA
Suggested value	0–3	> 0.900	>0.900	> 0.900	>0.900	> 0.900	< 0.080	<0.080
Value of this study	1.469	0.973	0.972	0.968	0.914	0.888	0.0483	0.043

**FIGURE 2:**
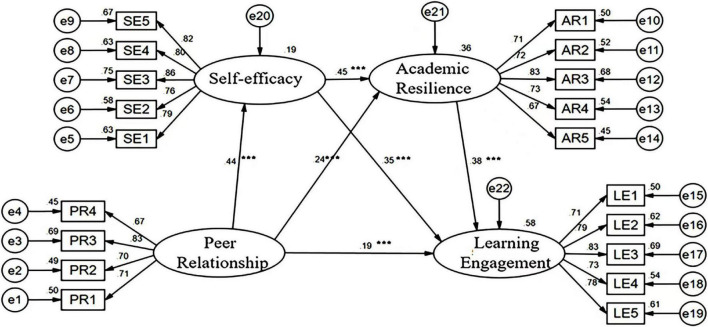
The structural modeling diagram. ***p < 0.001.

Most values reached the suggested value, indicating that the alternative structural model was revealed to be adequate. In addition, Figure 2 shows the explanatory variance and path coefficient of the alternative structural model with standardized parameter estimation. The construct of peer relationship explained 19% of the variance of the self-efficacy construct with a standardized regression coefficient of 0.437. The constructs of peer relationship and self-efficacy explained a 36% variance of academic resilience, with standardized regression coefficients of 0.244 and 0.450 respectively. Peer relationship, self-efficacy, and academic resilience illustrated a 58% variance of the learning engagement construct with the corresponding standardized regression coefficients of 0.193, 0.348, and 0.384 respectively. The bootstrap test was conducted with 5,000 resamplings, and all the path coefficients were statistically significant (*P* < 0.001). Therefore, the alternative structural model was verified by these data.

### Hypotheses tested

As shown in Table 6, the hypotheses H1, H2, H3, H5, H6, and H7 were statistically significant and their paths were supported by the empirical data. Specifically, peer relationship significantly and positively predicted learning engagement (β = 0.193, *P* < *0.01*), hence H1 was verified; peer relationship and self-efficacy established significant and positive relationships (β = 0.437, *P* < *0.001*), therefore H2 was supported; self-efficacy was significantly and positively related to learning engagement (β = 0.348, *P* < *0.001*), therefore H3 was verified; peer relationship was significantly and positively associated with academic resilience (β = 0.244, *P* < *0.01*), therefore H5 was supported; self-efficacy was significantly and positively correlated with academic resilience (β = 0.450, *P* < *0.001*), therefore H6 was verified; academic resilience significantly and positively predicted learning engagement (β = 0.384, *P* < *0.001*), therefore H7 was verified.

**TABLE 6:** The test results of path relationship.

Hypothesis	Path	Unstand estimates	Standard error	Z-value	Sig.	Stand estimates	Hypothesis test
H1	PR→LE	0.173	0.060	2.892	0.004	0.193	Supported
H2	PR→ SE	0.447	0.078	5.715	***	0.437	Supported
H3	SE→ LE	0.307	0.064	4.766	***	0.348	Supported
H5	PR→AR	0.230	0.071	3.212	0.001	0.244	Supported
H6	SE→AR	0.414	0.072	5.735	***	0.450	Supported
H7	AR→LE	0.367	0.075	4.918	***	0.384	Supported

PR, Peer Relationship; LE, Learning Engagement; SE, Self-efficacy; AR, Academic Resilience.

***p < 0.001.

### Analyses of the mediating effect of peer relationship on learning engagement

To analyze the mediating effect, the bootstrap method suggested by MacKinnon (2008) was used. It is believed that a statistically significant mediating effect must meet the following conditions: Z value is greater than 1.96 and the value of 95% bias-corrected confidence intervals (CI) excludes 0. As presented in Table 7, the total effect of peer relationship on learning engagement was 0.462 [Z = 5.250, 95% bias-corrected CI (0.307, 0.657), *P* < 0.01] and the direct effect of peer relationship on learning engagement was 0.173 [Z = 2.471, 95% bias-corrected CI (0.043, 0.316), *P* < 0.01], indicating that both the total effect and direct effect were statistically significant. The indirect effects were 0.068 [Z = 3.091, 95% bias-corrected CI (0.036, 0.132), *P* < 0.01] in the pathway of peer relationship-self-efficacy-academic resilience-learning engagement, 0.137 [Z = 2.978, 95% bias-corrected CI (0.067, 0.252), *P* < 0.01] in the pathway of peer relationship-self-efficacy-learning engagement, and 0.084 [Z = 2.270, 95% bias-corrected CI (0.028, 0.175), *P* < 0.01] in the pathway of peer relationship-academic resilience-learning engagement, showing that all the mediating effects were statistically significant.

**TABLE 7:** Direct, indirect and total effects of the hypothesized model.

Path relationship		Point estimate	Product of coefficient		Bootstrapping			
					Bias-corrected 95% CI		Percentile 95% CI	
					
			SE	Z-value	Lower	Upper	Lower	Upper
**Test of indirect, direct and total effects**								
DistalIE	PR→SE→AR→LE	0.068	0.022	3.091	0.036	0.132	0.032	0.120
SEIE	PR→SE→LE	0.137	0.046	2.978	0.067	0.252	0.063	0.245
ARIE	PR→AR→LE	0.084	0.037	2.270	0.028	0.175	0.024	0.166
TIE	Total indirect effect	0.289	0.055	5.255	0.198	0.412	0.196	0.407
DE	PR→LE	0.173	0.070	2.471	0.043	0.316	0.042	0.316
TE	Total effect	0.462	0.088	5.250	0.307	0.657	0.305	0.652
**Comparison of indirect effects**								
SEDIEdiff	SE VS.DistalIE	0.069	0.049	1.408	−0.013	0.186	−0.015	0.184
ARDIEdiff	AR VS.DistalIE	0.016	0.041	0.390	−0.070	0.105	−0.069	0.106
SEARdiff	SE VS. AR	0.053	0.069	0.768	−0.084	0.200	−0.083	0.203
**Percentage of indirect effects**								
P1	DistalIE/TIE	0.235	0.065	3.615	0.141	0.404	0.126	0.376
P2	SEIE/TIE	0.474	0.121	3.917	0.237	0.715	0.242	0.720
P3	ARIE/TIE	0.291	0.114	2.553	0.098	0.553	0.084	0.538
P4	TIE/TE	0.625	0.109	5.734	0.448	0.878	0.451	0.882
P5	DE/TE	0.375	0.109	3.440	0.122	0.552	0.118	0.549

To further explore the potential mediating roles played by self-efficacy and academic resilience in the association between peer relationship and learning engagement, three alternative models were tested. First, an alternative model was tested to examine the mediating role played by self-efficacy. In this case, the model was found to be adequate, with fit indices: X^2^/df = 1.591, IFI = 0.977, CFI = 0.976, TLI = 0.971, GFI = 0.937, AGFI = 0.910, SRMR = 0.0454, SMSEA = 0.049, indicating that self-efficacy played a mediating role in the association between peer relationship and learning engagement. Second, an alternative model was tested, in which academic resilience played a mediating role. The model was revealed to be adequate with fit indices: X^2^/df = 1.324, IFI = 0.985, CFI = 0.985, TLI = 0.982, GFI = 0.946, AGFI = 0.924, SRMR = 0.0405, SMSEA = 0.036, showing that academic resilience played a mediating role in the association between peer relationship and learning engagement. Third, an alternative model was tested to examine the mediating roles played by self-efficacy and academic resilience. The model was found to be adequate with fit indices (as shown in Table 5).

Data analysis indicated thatv the mediating effect of peer relationship on learning engagement was associated with self-efficacy and academic resilience, which significantly and positively played a partial mediating role in the association between peer relationship and learning engagement. And H4, H8, and H9 were also verified. In addition, the indirect effect percentage of self-efficacy and academic resilience as partial mediators were examined. As indicated in Table 7, the direct effect of peer relationship on learning engagement accounted for 37.5%, while the total indirect effect of peer relationship on learning engagement accounted for 62.5%, greater than the direct effect. Among the three significant indirect mediators, the indirect effect of self-efficacy is the greatest, accounting for 47.4% of the total indirect effect.

## Discussion

This study aimed to examine the association between peer relationship and learning engagement. In parallel, it also aimed to examine the mediating roles of self-efficacy and academic resilience in the association between peer relationship and learning engagement. The study tentatively proved that SCT can be used to explain the behaviors with regard to learning engagement. The findings are as follows.

Peer relationship is directly and positively associated with learning engagement which aligns with the research result of Juvonen et al. (2012) and Gremmen et al. (2018), that is, peer relationship contributes positively to learning engagement. One possible reason is that the classroom environment for peer interaction in school stimulates adolescents to improve their self-perception of efficacy, which is conducive to promoting learning engagement (Yang J. et al., 2021). In addition, peer relationship has been increasingly linked with different indicators of learning engagement (Ladd et al., 2009) and a stronger relationship with peers is related to higher classroom engagement (Fredricks et al., 2016). The results of this study further proved the prominent role of peer relationship in learning engagement.

Consistent with SCT, the results of the study identified self-efficacy as one significant partial mediating role on the pathway from peer relationship to learning engagement, which is consistent with previous studies (Sahil and Hashim, 2011; Gairns et al., 2015). Self-efficacy is concerned with individuals’ beliefs (Ritchie et al., 2021) and it is a premise of learning engagement (Shao and Kang, 2022). Students high in efficacy are more likely to show improvements in their effort and increase their engagement in learning activities (El-Sayad et al., 2021). The emergence of self-efficacy as a significant mediating role in the study further demonstrated the importance of self-efficacy in promoting adolescents’ learning engagement.

The results of the study demonstrated that academic resilience is another significant partial mediating variable, which is congruent with the suggestion of Permatasari et al. (2021) that the importance of resilience is highlighted between peer relationship and learning engagement. Students with high academic resilience can show flexibility and persistence when facing challenges and show more endeavor in overcoming difficulties, thus actively participating in learning (Ahmed et al., 2018). Similarly, the finding is consistent with another research result that peer interactions can be helpful in creating a soothing and supportive social environment that makes it possible for students to strengthen their academic resilience and stay engaged in learning (Gillham et al., 2013). In sum, the finding once indicated the role of academic resilience between peer relationship and learning engagement.

The results of the study also showed that self-efficacy and academic resilience functioned as a chain mediating role, which is one of the most striking findings. This means that self-efficacy and academic resilience sequentially played a mediating role in the association between peer relationship and learning engagement. The results of the study also revealed that among the three significant mediating roles, the mediating role of self-efficacy is the greatest, which is in line with the view that self-efficacy is the most important factor to change behaviors (Komendantova et al., 2018). In addition, the finding is similar to the result of Chu and Chu (2010) that self-efficacy plays the most important role in the relationship between peer support and learning engagement. Furthermore, it revealed that compared with peer relationship (β = 0.244, *P* < 0.01), adolescents’ self-efficacy contributed more to academic resilience (β = 0.450, *P* < 0.001). This may indicate that academic resilience was mainly derived from the self-efficacy of adolescents in the learning process due to their perceived ability to overcome difficulties in learning activities (Warshawski, 2022). Generally, the results of this study may enrich the research on learning behaviors to a certain extent by analyzing the complicated relations among peer relationship, self-efficacy, academic resilience, and learning engagement based on Social Cognitive Theory.

### The theoretical and practical implications

The study can make both theoretical and practical implications. Theoretically, this study has contributed to the literature in two aspects. On one hand, the findings of this study indicate that the peer relationship has a positive impact on learning engagement, which may offer extended knowledge in understanding the mechanism between peer relationship and learning engagement. Specifically, individuals who can get support from their peers may change their learning behaviors and improve their learning engagement (Kiefer et al., 2015). On the other hand, the study has shown that the mediating roles of self-efficacy and academic resilience may explain how peer relationship is associated with learning engagement, which enriches the literature about learning engagement (Juvonen et al., 2012; Gremmen et al., 2018; Fredricks et al., 2019). The study tentatively proves that self-efficacy and academic resilience can significantly transmit the positive impact of peer relationship on learning engagement. In learning, adolescents with stronger self-efficacy and academic resilience will hold better psychological state of mind concerning learning behaviors. Learning context with positive peer relationship can foster adolescents’ personal factors—self-efficacy and academic resilience, which in turn facilitates their learning engagement. Practically, the study can help educational practitioners understand students’ learning engagement better from the perspective of environmental aspect (e.g., peer relationship) and learner factors such as self-efficacy and academic resilience. Concerning peer relationship, adolescents should be provided with necessary training, lectures, and symposiums that may help them realize the importance of developing sound peer relationship and improve their skills in building friendships with peers (Doumen et al., 2012). Besides, adolescents’ group work and cooperation with peers should be strengthened in learning contexts so as to promote their learning engagement (Yang J. et al., 2021). In terms of self-efficacy, strategies should be offered to help adolescents develop self-efficacy and approach their learning actively. In addition, adolescents’ confidence should be enhanced through educational programs to make them get over any difficulties in learning activities. With regard to academic resilience, teachers should develop adolescents’ strategies and skills to enhance their persistence and flexibility through purposeful projects and activities in classroom teaching and/or relevant training programs.

### Limitations and future research directions

Limitations in the study should be stated. First, the proposed theoretical model was tested only in connection with the sample selected from one school, which may limit the generalizability of the findings. Further validation of the model with diverse samples from more schools is needed in the future. Second, this study explores the mechanism between peer relationship and learning engagement with the mediating roles of self-efficacy and academic resilience. However, there are more factors affecting learning engagement, such as academic stress, learning motivation, self-assessment, and so on. Future studies should take more variables into consideration so as to derive more convincing results and suggestions for practice. Third, the study focused on the cross-sectional study design, so it may make us unable to infer causal relations among the variables. Future studies could focus on longitudinal studies to explore the relationship between peer relationship and learning engagement.

## Data availability statement

The original contributions presented in this study are included in the article/Supplementary material, further inquiries can be directed to the corresponding author.

## Author contributions

YS analyzed the data and wrote the manuscript. SK developed the conceptual framework and revised the manuscript. Both authors contributed to the article and approved the submitted version.
